# Fabrication and Characterization of a CMOS-MEMS Humidity Sensor 

**DOI:** 10.3390/s150716674

**Published:** 2015-07-10

**Authors:** John-Ojur Dennis, Abdelaziz-Yousif Ahmed, Mohd-Haris Khir

**Affiliations:** 1 Department of Fundamental and Applied Sciences, Universiti Teknologi PETRONAS, Bandar Seri Iskandar, Perak Darul Ridzuan 32610, Malaysia; E-Mail: johndennis@petronas.com.my; 2 Department of Electrical and Electronic Engineering, Universiti Teknologi PETRONAS, Bandar Seri Iskandar, Perak Darul Ridzuan 32610, Malaysia; E-Mail: harisk@petronas.com.my

**Keywords:** CMOS-MEMS humidity sensor, titanium dioxide nanoparticles, post-CMOS micromachining, sensor response, recovery and repeatability

## Abstract

This paper reports on the fabrication and characterization of a Complementary Metal Oxide Semiconductor-Microelectromechanical System (CMOS-MEMS) device with embedded microheater operated at relatively elevated temperatures (40 °C to 80 °C) for the purpose of relative humidity measurement. The sensing principle is based on the change in amplitude of the device due to adsorption or desorption of humidity on the active material layer of titanium dioxide (TiO_2_) nanoparticles deposited on the moving plate, which results in changes in the mass of the device. The sensor has been designed and fabricated through a standard 0.35 µm CMOS process technology and post-CMOS micromachining technique has been successfully implemented to release the MEMS structures. The sensor is operated in the dynamic mode using electrothermal actuation and the output signal measured using a piezoresistive (PZR) sensor connected in a Wheatstone bridge circuit. The output voltage of the humidity sensor increases from 0.585 mV to 30.580 mV as the humidity increases from 35% RH to 95% RH. The output voltage is found to be linear from 0.585 mV to 3.250 mV as the humidity increased from 35% RH to 60% RH, with sensitivity of 0.107 mV/% RH; and again linear from 3.250 mV to 30.580 mV as the humidity level increases from 60% RH to 95% RH, with higher sensitivity of 0.781 mV/% RH. On the other hand, the sensitivity of the humidity sensor increases linearly from 0.102 mV/% RH to 0.501 mV/% RH with increase in the temperature from 40 °C to 80 °C and a maximum hysteresis of 0.87% RH is found at a relative humidity of 80%. The sensitivity is also frequency dependent, increasing from 0.500 mV/% RH at 2 Hz to reach a maximum value of 1.634 mV/% RH at a frequency of 12 Hz, then decreasing to 1.110 mV/% RH at a frequency of 20 Hz. Finally, the CMOS-MEMS humidity sensor showed comparable response, recovery, and repeatability of measurements in three cycles as compared to a standard sensor that directly measures humidity in % RH.

## 1. Introduction

A relative humidity sensor is a form of chemical sensor that responds to the presence of water molecules in the environment. Humidity is the amount of water vapor in the air compared with the amount of vapor needed to make the air saturated at its current temperature. Humidity sensors can be used in many applications such as medical equipment, agriculture, heating, ventilation and air conditioning (HVAC) systems, the automobile and semiconductor industry, intelligent control of the living environment in buildings, hygrometers and consumer goods, and cooking food control for microwave ovens [1]. Fenner *et al.* [2] reported that indoor relative humidity should be between 35% RH and 65% RH for the comfort of the occupants. Relative humidity below or above this range causes discomfort as well as health problems such as chapped lips, bleeding nose, and dry throat. Therefore, humidity sensors are very important in our daily life. 

### 1.1. MOS and Conducting Polymer Based Humidity Sensor 

Traditional humidity sensors depend on changes in electrical properties of thin films of various materials; these changes can be estimated by measuring either a resistance [3,4,5,6,7] or a capacitance [8,9,10,11,12,13] change of the thin films. These types of humidity sensors may be divided into two categories: metal oxide semiconductor MOS- and polymer-based humidity sensors. In recent years, there have been many published research papers on MOS-based humidity sensors [14,15,16]; tin dioxide (SnO_2_) and titanium dioxide (TiO_2_) are very popular sensing materials for these types of humidity sensors. Polymer-based sensors have also been used for humidity detection for a very long time [17,18,19].

### 1.2. CMOS- and CMOS-MEMS-Based Humidity Sensors

Humidity sensors of these types are fabricated using standard CMOS process with some form of post-CMOS micromachining or other additional post-CMOS processes to complete the sensor design. These process technologies provide enhancement in the size and cost due to the possibility of batch fabrication. These types of humidity sensors may be divided into two categories: CMOS- and CMOS-MEMS-based humidity sensors.

### 1.3. CMOS-Based Humidity Sensors

Miniaturization trends have necessitated the fabrication of resistive or capacitive MOS- or polymer-based humidity sensors using CMOS process technology and some additional post-CMOS steps such as drop-coating or deposition of sensitive materials on the CMOS die. Their working principle is similar to MOS- and polymer-based sensors in that they convert information about the humidity level in the air into electrical signals [11]. The operation of the sensors is based on interaction between the water molecules in the air (absorption or adsorption) and the sensitive material layer, which induces a change in its electrical properties such as resistance or capacitance changes. The changes in the sensitive layer are detected by the respective transducer and translated into current or voltage output. The design and fabrication of different types of CMOS-based humidity sensors have been reported by many researchers [10,20,21].

### 1.4. CMOS-MEMS-Based Humidity Sensors

MEMS is a technology that integrates mechanical elements, sensors, actuators, and electrical and electronic components on a common silicon substrate with feature sizes ranging from millimeters to micrometers. The most significant advantage of MEMS is their ability to communicate easily with electrical elements in semiconductor chips. Furthermore, there are many other advantages for MEMS such as small size, low power consumption, low cost, easy integration into systems, and possibility of use for array fabrication. The scaling down of micromechanical to micromechanical devices provides an improvement in several areas as demonstrated in applications such as inertial sensors, ink-jet printers, RF communications, satellites, smart phones, pressure sensors, accelerometers, gyroscopes, bio-medical devices, military systems, and chemical sensors [9,22,23,24]. When CMOS layers are used in MEMS devices as structural layers and post-CMOS micromachining is used to release these structures, the resulting devices are known as CMOS-MEMS devices [17,25,26]. The design and fabrication of different types CMOS-MEMS humidity sensors have been reported by many researchers [9,11,17,25]. 

Some of the primary mechanical elements utilized in the development of CMOS-MEMS devices included micro-sized cantilevers and membranes or plates. Such devices are often used in sensing or actuation technologies and are generally based upon the changes in physical properties such as mass or stress of the device due to absorption/adsorption of gas species at the surface. Mass changes can be detected by monitoring frequency changes of the resonating structure or observing the deflection of the micromechanical structure due to mass loading and surface stress changes (static mode) [27]. Mass-sensitive (gravimetric) sensors have been used for humidity sensing by detecting the changes in their resonant frequency due to mass change [28,29,30,31,32,33]. 

Many of the current CMOS-MEMS resonant microstructures used as humidity sensors have a sensitive layer of polymer coating and are usually operated at room temperature. However, the humidity absorbed in the detection process in many instances adheres onto the polymer and cannot be easily desorbed. These characteristics cause problems in real-time measurement where humidity levels are changing and results in hysteresis when measurements are done in an environment with increasing and decreasing humidity levels. Many studies have been conducted to investigate hysteresis and reported hysteresis as high as 6.6% RH at 25 °C ambient temperature[21], while another research reported 1.3% RH at 56% RH for absorption and desorption [9] and 1.6% RH in a range from 40% RH to 90% RH has been presented by Lee *et al.* [4]. Therefore, there is a need to investigate and develop technologies to improve reversibility, stability, and hysteresis of humidity sensors.

This paper reports the fabrication and characterization of a CMOS-MEMS humidity sensor with embedded microheater element that is employed for electrothermal actuation of the device as well as for maintaining the device plate at a moderately elevated temperature of 40 °C to 80 °C in order to improve reversibility, stability, and hysteresis in humidity detection.

## 2. Fabrication and Characterization of the CMOS-MEMS Humidity Sensor

### 2.1. Fabrication of the Sensor

**Figure 1:**
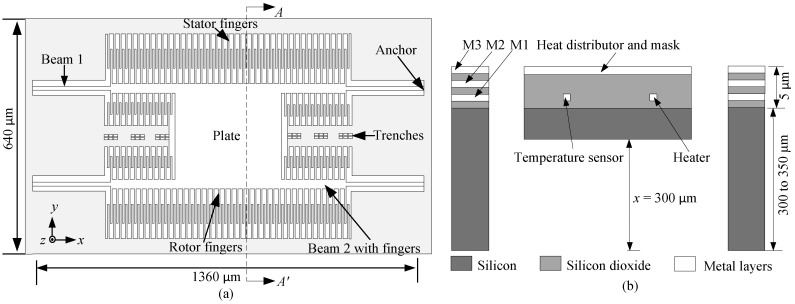
(**a**) Schematic of the top view; (**b**) Cross-sectional view along AA’ showing CMOS layers and the SCS substrate.

The sensor device is fabricated using standard 0.35 µm CMOS process technology and post-CMOS micromachining through a multiple project wafer (MPW). The CMOS process technology consists of two polysilicon, three metal, several dielectric layers, and two vias. All metal layers are aluminum (Al), whereas silicon dioxide (SiO_2_) is used as the dielectric layer and tungsten is used for the vias. Figure 1a shows a schematic of the top view of the device and consists of a plate supported by four beams with each beam having two sections of different widths of 7 µm and 19 µm but the same length of 240 µm each. The stator comb-fingers are anchored to the substrate while the rotor comb-fingers are attached to the 400 µm × 400 µm plate and also to the thick 19 µm beam (beam 2). Figure 1b shows a schematic cross-sectional view of the device viewed along AA’ to show the CMOS layers and the single crystal silicon (SCS) substrate.

**Figure 2:**
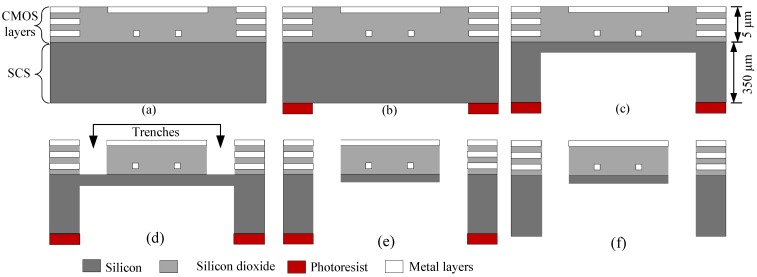
Schematic cross-sectional view of (**a**) Back grind; (**b**) PR layer deposited; (**c**) Anisotropic etching of Si from backside; (**d**) Anisotropic etching of SiO_2_ from front side; (**e**) Isotropic etching of Si from front side; (**f**) Released device with photoresist mask removed.

After completion of the CMOS process, before the dry etching step, the eight-inch wafer is back-grinded to a thickness of 350 µm from the initial thickness of 750 µm, as shown in the cross section view in Figure 2a. A thick photoresist (PR) layer is then coated on the backside of the eight-inch wafer and patterned as shown in Figure 2b to expose the regions of the sensor devices that need to be exposed while protecting the bonding pads and other regions of the CMOS-MEMS devices that do not need to be released. Anisotropic Deep Reactive Ion Etching (DRIE) is then used to partially etch the SCS substrate from the backside, leaving a layer of 15 µm thick SCS under the CMOS layers to define the MEMS structures as shown in Figure 2c. After SCS etching from backside, the wafer is diced into individual chips and the chips are then mounted on a carrier wafer for front side etching. Figure 2d shows the appearance of trenches after front side etching of SiO_2_ using Reactive Ion Etching (RIE), while Figure 2e shows a released MEMS structure after using isotropic DRIE to etch through Si from front side. Finally, the photoresist mask is removed to complete the post-CMOS micromachining process as shown in Figure 2f.

### 2.2. Characterization Setup for the CMOS-MEMS Humidity Sensor

**Figure 3:**
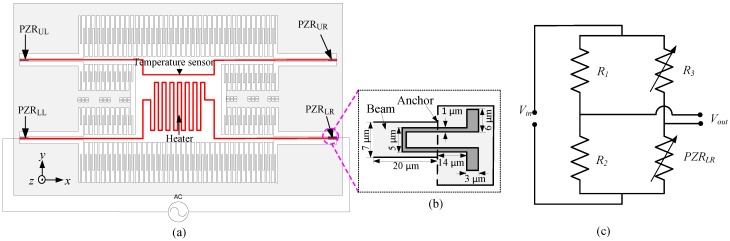
(**a**) Schematic of electrothermal actuation method and piezoresistive sensing; (**b**) A magnified view of PZR_LR_; (**c**) Wheatstone bridge circuit for measurement of the output signal.

The CMOS-MEMS device is operated in the dynamic mode using electrothermal actuation by applying an AC-current through the embedded microheater as shown in Figure 3a to produce oscillations of the plate in the out-of-plan *z* direction. These vibrations produce stress at the anchor points of the four supporting beams and this stress changes the resistance of the PZRs, which can be measured using a Wheatstone bridge circuit. In the initial experimental setup on which the results of this paper are based, a single longitudinal PZR_LR_ (where the subscript LR stands for the lower right hand side of the device) is connected to three external resistors, *R_1_*, *R_2_*, and *R_3_* of the same values as those of the PZR to construct a Wheatstone bridge circuit configuration. One of the three external resistors, *R_3_*, is a variable resistor to adjust the Wheatstone bridge circuit. Figure 3b shows a magnified view of the PZR_LR_, while Figure 3c shows the Wheatstone bridge circuit configuration to convert changes in the resistance of the PZR_LR_ to voltage output *V_out_* and is biased using a DC voltage input *V_in_* of 3 V.

**Figure 4:**
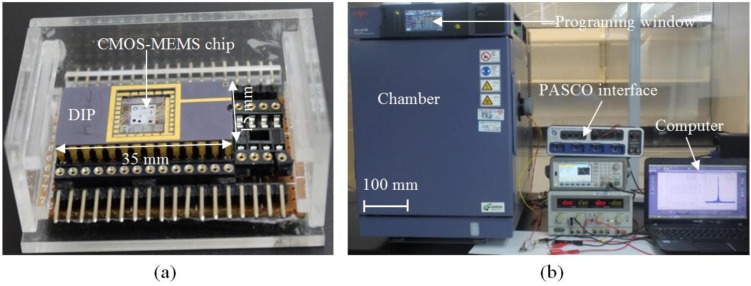
(**a**) Bonded and packaged device; (**b**) Experimental setup for measurement of the humidity response of the CMOS-MEMS device.

The sensor device that was bonded and packaged in a commercial dual in line package (DIP) as shown in Figure 4a was placed on a sample holder inside a bench-top type SH-242 temperature and humidity chamber of size 300 × 300 × 250 mm, as shown in Figure 4b. For the first experiment, the CMOS-MEMS humidity sensor inside the chamber is operated in the dynamic mode by applying an input AC voltage at frequency of 4 Hz with amplitude of 6 V_pp_. The relative humidity inside the chamber is increased and decreased from 35% RH to 95% RH in steps of 5% RH while keeping the ambient temperature constant at 27 °C to observe hysteresis. The time for a 5% RH step increase in humidity is set to 7 min and, in between each step, the humidity is maintained constant for 3 min before increasing it to the next level in another 7 min; the output voltage is measured using PASCO interface card (PS-2124A) connected to a computer, as shown in Figure 4b.

In order to understand the influence of temperature, the device is driven in the dynamic mode by applying a 2 Hz AC signal with different applied voltages of 2, 3, 4, 5 and 6 V_pp_ corresponding to different operating temperatures of 40, 50, 60, 70 and 80 °C, respectively, as determined from Temperature Coefficient of Resistance (TCR) measurements [34], while the relative humidity is varied from 60% RH to 95% RH in steps of 5% RH. To investigate the effect of operating the sensor device at different input frequencies on its sensitivity, the device is electrothermally driven in the dynamic mode by applying different input frequencies from 2 Hz to 20 Hz with amplitude of 6 V_pp_ for the same range of relative humidity from 60% RH to 95% RH. 

The response, recovery, and repeatability of the sensor in the chamber were also measured together with a standard humidity sensor (PASPORT Humidity/Temp/Dew Point Sensor PS-2124A) that is used for validation of the results. The device was again operated in the dynamic mode by applying a 2 Hz AC signal with amplitude of 5 V_pp_ and the relative humidity inside the chamber was programmed to change from ambient humidity of 68% RH to 95% RH. Once the humidity inside the chamber reaches the 95% RH set point, the chamber door is then abruptly opened, resulting in an abrupt drop in humidity level from 95% RH to the ambient humidity. Next, the chamber was closed again and the humidity inside the chamber allowed to increase from the ambient to 95% RH. These experimental steps were repeated three times in order to observe the response, recovery, repeatability, and stability of the sensor and the data was continuously recorded in a computer.

## 3. Results and Discussion

**Figure 5:**
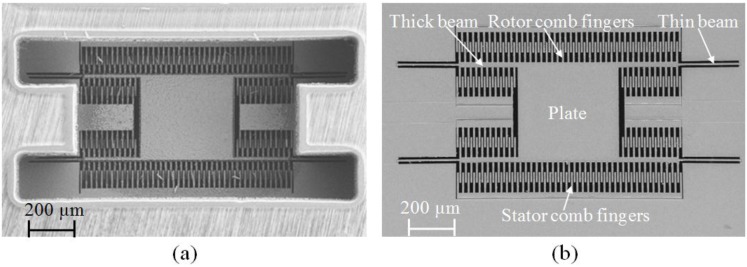
FESEM image of the CMOS-MEMS device from (**a**) Backside; (**b**) Front side [35].

Figure 5a shows a Field Emission Scanning Electron Microscope (FESEM) image of the backside of the device, indicating the successful etching of the SCS leaving approximately 15 μm SCS to define the MEMS structure. Figure 5b shows the front side of the successfully released device.

**Figure 6:**
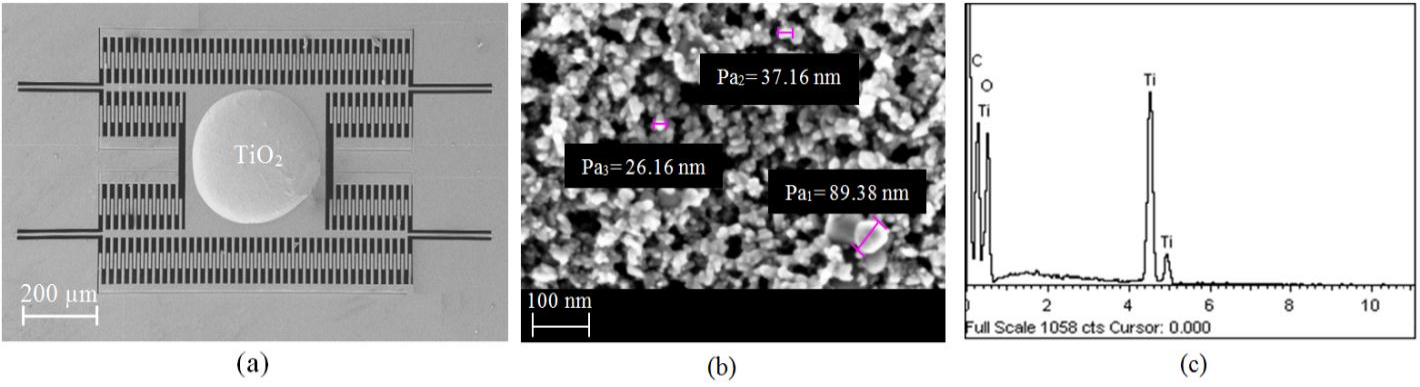
(**a**) FESEM image of CMOS-MEMS device with TiO_2_ paste deposited on its plate; (**b**) Zoomed-in FESEM micrograph of TiO_2_ nanoparticles. (**c**) EDX-spectrum of the TiO_2_ on the plate.

Figure 6a shows an FESEM image of the CMOS-MEMS humidity sensor with nanoparticle-sized TiO_2_ paste successfully deposited on its plate using a drop-coating method; Figure 6b is a zoomed-in view of an FESEM micrograph on the TiO_2_ patch on the plate showing the porous distribution of nanoparticles of TiO_2_; and Figure 6c shows EDX-spectrum of TiO_2_. The carbon peak observed in the EDX spectrum is from the binder material used to form the paste. The porous structure of TiO_2_ on the plate increases its surface area and thus is expected to enhance its sensitivity in the adsorption/desorption reaction of H_2_O on its surface.

**Figure 7:**
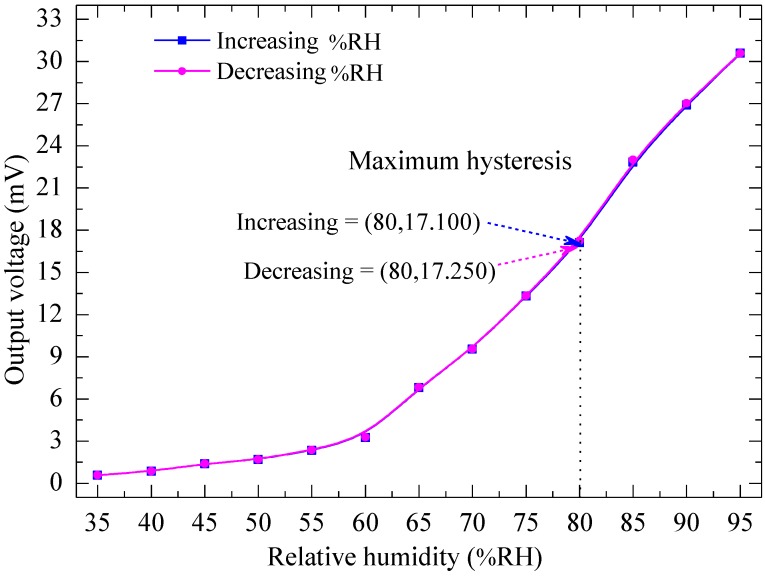
Output voltage *vs.* relative humidity at an operating temperature of 80 °C, corresponding to applied voltages of 6 V_pp_ at a frequency of 4 Hz.

**Figure 8:**
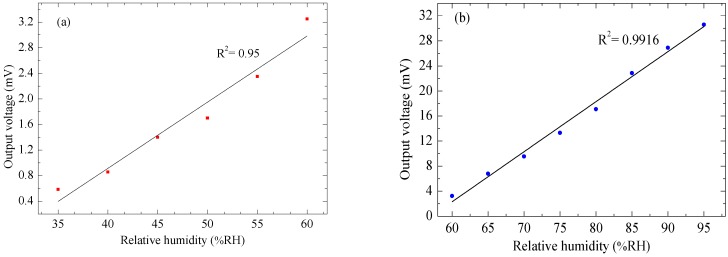
Output voltage of the CMOS-MEMS humidity sensor *vs.* changes in relative humidity level from (**a**) 35% RH to 60% RH; (**b**) 60% RH to 95% RH.

Figure 7 shows the output voltage of the device for increasing and decreasing humidity levels from 35% RH to 95% RH in steps of 5% RH when the device was operated at a frequency of 4 Hz at an applied voltage of 6 V_pp_, corresponding to a temperature on the plate of 80 °C. The maximum hysteresis is found to be 0.87% RH at 80% RH, indicating comparatively low hysteresis, which gives the device a high advantage for use in real-time measurements. The output voltage of the humidity sensor increases from 0.585 mV to 30.580 mV as the humidity is increased from 35% RH to 95% RH. It was observed to be linear from 0.585 mV to 3.250 mV as the humidity increases from 35% RH to 60% RH with sensitivity of 0.107 mV/% RH as shown in Figure 8a, and again linear from 3.250 mV to 30.580 mV as the humidity level increases from 60% RH to 95% RH with sensitivity of 0.781 mV/% RH as shown in Figure 8b. 

**Figure 9:**
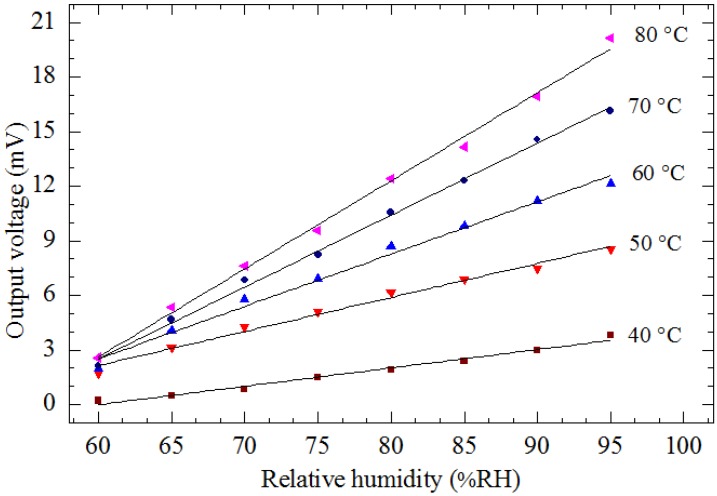
Output voltage *vs.* relative humidity at various applied voltages from 2 V_pp_ to 6 V_pp_ (corresponding to different operating temperatures from 40 °C to 80 °C) at a constant frequency of 2 Hz.

**Figure 10:**
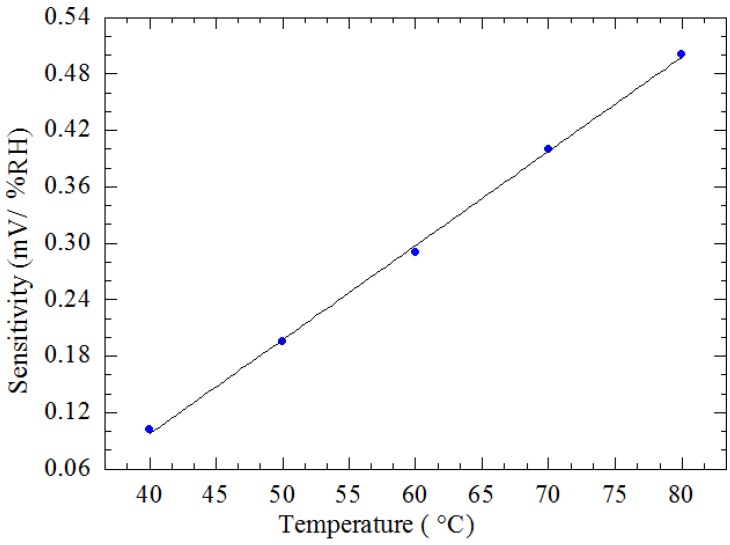
Sensitivity of the CMOS-MEMS humidity sensor *vs.* temperature.

Figure 9 shows the measured results of output voltage of the device as a function of the relative humidity in the linear region from 60% RH to 95% RH when the device was operated at various applied voltages from 2 V_pp_ to 6 V_pp_ (corresponding to different operating temperatures from 40 °C to 80 °C) at a constant frequency of 2 Hz. It is observed that the output voltage of the device increases with increasing humidity for different operating temperatures. The slopes of the graph indicate that the sensitivity improves at higher temperatures, as indicated in Figure 10, where the sensitivity is seen to increase linearly from 0.102 mV/% RH to 0.501 mV/% RH with an increase in the temperature from 40 °C to 80 °C. This increase in sensitivity with temperature is expected as desorption of gaseous species such as H_2_O in humidity detection on the TiO_2_ surface is enhanced with increase in temperature. At higher temperatures more gaseous species desorb from the sensor surface, resulting in an overall decrease in the mass of the plate and TiO_2_ and thus an increase in the amplitude of the vibrating structure.

**Figure 11:**
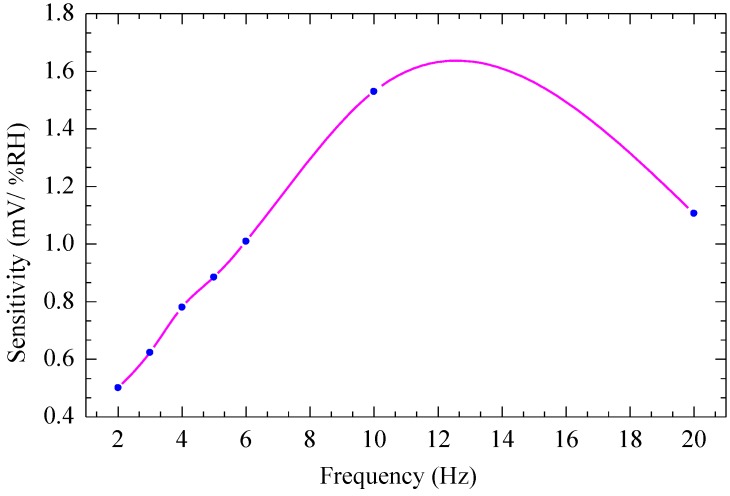
Sensitivity of the CMOS-MEMS humidity sensor *vs.* frequency.

Figure 11 shows the experimental results of sensitivity of the CMOS-MEMS humidity sensor as a function of the applied frequency. The device is operated by applying various frequencies from 2 Hz to 20 Hz with amplitude of 6 V_pp_ at relative humidity from 60% RH to 95% RH. It is observed that the sensitivity of the device increases from 0.500 mV/% RH to reach a maximum value of 1.634 mV/% RH at a frequency of 12 Hz, then decreases to 1.110 mV/% RH at frequency of 20 Hz. The observed maximum at the frequency of 12 Hz is assumed to be due to the effect of air damping, which increases as the frequency increases. This increase in air damping affects the driving force, which results in decreased amplitude after a certain optimal frequency is reached (about 12 Hz in this case). 

**Figure 12:**
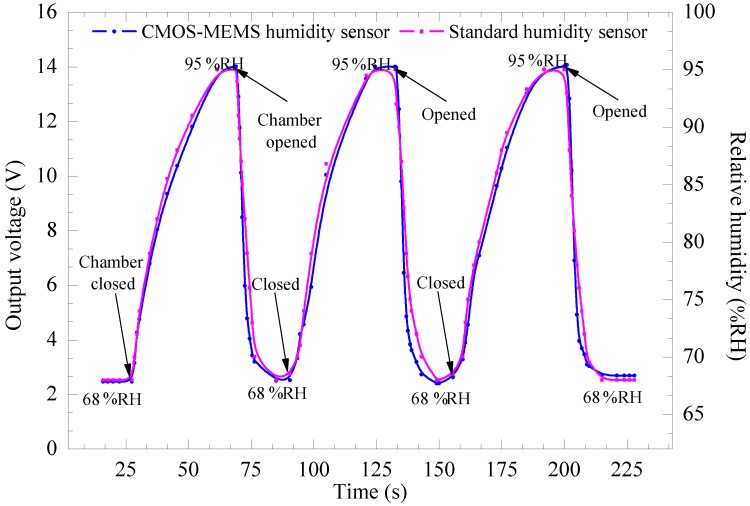
Response, recovery, and repeatability of the device and standard humidity sensor *vs.* time for three cycles at operating frequency of 2 Hz at 5 V_pp_.

Figure 12 shows experimental results of response, recovery, and repeatability for the device as compared to a standard humidity sensor when the device was operated in the dynamic mode by applying a 2 Hz AC signal with amplitude of 5 V_pp_ and the relative humidity varied from ambient humidity of 68% RH to 95% RH for three cycles of measurement. It is observed that the output voltage of the CMOS-MEMS humidity sensor follows the standard sensor that directly measures humidity in % RH with a good comparable response, recovery, and stable repeatability. 

## 4. Conclusions

A CMOS-MEMS device with embedded microheater for relative humidity sensing via amplitude change, due to mass loading, has been fabricated and characterized. The device was designed and fabricated using 0.35 µm CMOS process technology and post-CMOS micromachining. The device was operated in dynamic mode using electrothermal actuation and piezoresistive sensing was used to measure the output voltage via a Wheatstone bridge circuit. Nanostructured TiO_2_ paste was deposited on the plate of the CMOS-MEMS humidity sensor as the active sensing element. Results showed that the output voltage of the humidity sensor increased from 0.585 mV to 30.580 mV as the humidity increased from 35% RH to 95% RH. It was observed that the output voltage was linear from 35% RH to 60% RH with sensitivity of 0.107 mV/% RH, and from 60% RH to 95% RH with a higher sensitivity of 0.781 mV/% RH; the maximum hysteresis was found to be 0.87% RH at 80% RH. Furthermore, the sensitivity of the humidity sensor increased linearly from 0.102 mV/% RH to 0.501 mV/% RH with an increase in the temperature from 40 °C to 80 °C; it was also found to be dependent on the frequency of driving voltage with a maximum sensitivity observed at the frequency of 12 Hz. Finally, the CMOS-MEMS humidity sensor showed a comparable response, recovery, and repeatability of measurements as compared to a standard sensor that directly measures humidity in % RH. 

## References

[1] Atta N.F. (2013). Nanosensors: Materials and Technologies.

[2] Fenner R., Zdankiewicz E. (2001). Micromachined Water Vapor Sensors: A Review of Sensing Technologies. IEEE Sens. J..

[3] Sreelekshmi M., Gupta S., Chidambaram K. (2013). Room Temperature Synthesized Nano Metal Oxide Humidity Sensor. Inter. J. Appl. Eng. Res..

[4] Lee D.H., Hong H.K., Park C.K., Kim G.H., Jeon Y.S., Bu J.U. A Micromachined Robust Humidity Sensor for Harsh Environment Applications. Proceedings of the 14th IEEE International Conference on Micro Electro Mechanical Systems (MEMS).

[5] Yang M.Z., Dai C.L., Lin W.Y. (2011). Fabrication and Characterization of Polyaniline/PVA Humidity Microsensors. Sensors.

[6] Lee C.W., Gong M.S. (2003). Resistive Humidity Sensor Using Phosphonium Salt-Containing Polyelectrolytes Based on the Mutually Cross-Linkable Copolymers. Macromol. Res..

[7] Kiasari N.M., Soltanian S., Gholamkhass B., Servati P. (2012). Room Temperature Ultra-Sensitive Resistive Humidity Sensor Based on Single Zinc Oxide Nanowire. Sens. Actuators A Phys..

[8] Lee M.J., Lee C.J., Singh V., Yoo K.P., Min N.K. Humidity Sensing Characteristics of Plasma Functionalized Multiwall Carbon Nanotube-polyimide Composite Films. Proceedings of the IEEE Sensors.

[9] Kim J.H., Hong S.M., Lee J.S., Moon B.M., Kim K. High Sensitivity Capacitive Humidity Sensor with a Novel Polyimide Design Fabricated by MEMS Technology. Proceedings of the 4th IEEE International Conference on Nano/Micro Engineered and Molecular Systems (NEMS).

[10] Gu L., Huang Q.A., Qin M. (2004). A Novel Capacitive-Type Humidity Sensor Using CMOS Fabrication Technology. Sens. Actuators B Chem..

[11] Dai C.L. (2007). A Capacitive Humidity Sensor Integrated with Micro Heater and Ring Oscillator Circuit Fabricated by CMOS-MEMS Technique. Sens. Actuators B Chem..

[12] Kim J.H., Hong S.M., Moon B.M., Kim K. (2010). High-Performance Capacitive Humidity Sensor with Novel Electrode and Polyimide Layer Based on MEMS Technology. Microsys. Technol..

[13] Kim J.H., Moon B.M., Hong S.M. (2012). Capacitive Humidity Sensors Based on a Newly Designed Interdigitated Electrode Structure. Microsys. Technol..

[14] Hu Y.C., Dai C.L., Hsu C.C. (2014). Titanium Dioxide Nanoparticle Humidity Microsensors Integrated with Circuitry on-a-Chip. Sensors.

[15] Shukla S.K., Bharadvaja A., Parashar G.K., Mishra A.P., Dubey G.C., Tiwari A. (2012). Fabrication of ultra-sensitive optical fiber based humidity sensor using TiO_2_ thin film. Adv. Mater. Lett..

[16] Dokmeci M., Najafi K. (2001). A high-sensitivity polyimide capacitive relative humidity sensor for monitoring anodically bonded hermetic micropackages. J. Microelectromech. Syst..

[17] Lazarus N., Bedair S.S., Lo C.C., Fedder G.K. (2010). CMOS-MEMS Capacitive Humidity Sensor. J. Microelectromech. Syst..

[18] Bedair S.S., Fedder G.K. Polymer Mass Loading of CMOS/MEMS Microslot Cantilever for Gravimetric Sensing. Proceedings of the IEEE Sensors.

[19] Bedair S.S., Fedder G.K. Polymer Wicking to Mass Load Cantilevers for Chemical Gravimetric Sensors. Proceedings of the 13th International Conference on Solid-State Sensors, Actuators and Microsystems.

[20] Deng F., He Y., Zhang C., Feng W. (2014). A CMOS Humidity Sensor for Passive RFID Sensing Applications. Sensors.

[21] Nizhnik O., Higuchi K., Maenaka K. (2011). A Standard CMOS Humidity Sensor without Post-Processing. Sensors.

[22] Zalalutdinov M.K., Cross J.D., Baldwin J.W., Ilic B.R., Zhou W., Houston B.H., Parpia Jeevak, M. (2010). CMOS-Integrated RF MEMS Resonators. J. Microelectromech. Sys..

[23] Yusof N.B., Soin N., Dawal S.Z.M. Capacitive Interfacing for MEMS Humidity and Accelerometer Sensors. Proceedings of the International Conference for Technical Postgraduates (TECHPOS).

[24] Khir M.H.M., Qu P., Qu H.W. (2011). A Low-Cost CMOS-MEMS Piezoresistive Accelerometer with Large Proof Mass. Sensors.

[25] Yang T.Y., Huang J.J., Liu C.Y., Wang H.Y. A CMOS-MEMS Humidity Sensor. Proceedings of the International Conference on Circuits, System and Simulation (ICCSS).

[26] Saha T. (2012). Design, Fabrication, and CMOS Integration of MEMS Humidity Sensors. Master’s Thesis.

[27] Hierlemann A., Brand O., Hagleitner C., Baltes H. (2003). Microfabrication Techniques for Chemical/Biosensors. IEEE Proc..

[28] Lei S., Chen Y., Li Y. A Novel SAW Humidity Sensor Based on Electrosprayed Polymerized Electrolyte Film. Proceedings of the 2011 Third International Conference on Measuring Technology and Mechatronics Automation (ICMTMA).

[29] Pascal-Delannoy F., Sorli B., Boyer A. (2000). Quartz Crystal Microbalance (QCM) Used as Humidity Sensor. Sens. Actuators A Phys..

[30] Fragakis J., Chatzandroulis S., Papadimitriou D., Tsamis C. (2005). Simulation of Capacitive Type Bimorph Humidity Sensors. J. Phys. Conf. Ser..

[31] Nuryadi R., Djajadi A., Adiel R., Aprilia L., Aisah N. (2013). Resonance Frequency Change in Microcantilever-Based Sensor due to Humidity Variation. Mater. Sci. Forum.

[32] Sappat A., Wisitsoraat A., Sriprachuabwong C., Jaruwongrungsee K., Lomas T., Tuantranont A. Humidity Sensor Based on Piezoresistive Microcantilever with Inkjet Printed PEDOT/PSS Sensing Layers. Proceedings of the 8th International Conference on Electrical Engineering/Electronics, Computer, Telecommunications and Information Technology (ECTI-CON).

[33] Kima B.H., Prinsa F.E., Kerna D.P., Raibleb S., Weimarb U. (2001). Multicomponent Analysis and Prediction with a Cantilever Array Based Gas Sensor. Sens. Actuators B Chem..

[34] Ahmed A.Y., Dennis J.O., Khir M.H.M., Saad M.N.M. Design and Characterization of Embedded Microheater on CMOS-MEMS Resonator for Application in Mass-Sensitive Gas Sensors. Proceedings of the 2014 5th International Conference on Intelligent and Advanced Systems: Technological Convergence for Sustainable Future (ICIAS).

[35] Dennis J.O., Ahmed A.Y., Khir M.H.M., Rabih A.A.S. (2015). Modelling and Simulation of the Effect of Air Damping on the Frequency and Quality factor of a CMOS-MEMS Resonator. Appl. Math. Infor. Sci. (AMIS).

